# Two cases of diabetic macular edema complicated by an atypical macular hole

**DOI:** 10.1186/s12886-020-01444-7

**Published:** 2020-04-29

**Authors:** Yuich Yoshida, Takaki Sato, Shou Oosuka, Masashi Mimura, Masanori Fukumoto, Takatoshi Kobayashi, Teruyo Kida, Tsunehiko Ikeda

**Affiliations:** Department of Ophthalmology, Osaka Medical College, 2-7 Daigaku-machi, Takatsuki-City, Osaka, 569-8686 Japan

**Keywords:** Macular hole (MH), Diabetic macular edema (DME), Pars plana vitrectomy (PPV), Retinoschisis (RS), Serous retinal detachment (SRD)

## Abstract

**Background:**

Here we report two patients who developed an atypical macular hole (MH) during the treatment course for diabetic macular edema (DME).

**Case presentations:**

Patient 1 was a 73-year-old male. Optical coherence tomography (OCT) revealed perifoveal retinoschisis (RS) in addition to cystoid macular edema and serous retinal detachment (SRD) in his left eye, and that an MH had developed during the clinical course. A convex surface was formed at the MH margin toward the vitreous cavity, and granular shadows were observed in the fluid cuff. Intraoperative findings revealed a thin epiretinal macular membrane (ERM) around the MH. Patient 2 was a 79-year-old male. Although the patient underwent pars plana vitrectomy (PPV) for proliferative diabetic retinopathy (PDR) in both eyes, RS and a thin ERM in addition to SRD was observed in his left eye after surgery, and an MH developed during the clinical course. As in Patient 1, a convex surface was formed at the fluid cuff margin toward the vitreous cavity.

**Conclusions:**

Both patients had persistent DME, SRD, RS, and a thin ERM before the development of the MH. OCT revealed the formation of a convex surface at the MH margin toward the vitreous cavity, suggesting that the fragility of the layered structure of the retina combined with tangential retinal traction may have been involved in the atypical MH form.

## Background

Treatment for diabetic macular edema (DME) includes the topical administration of steroids and anti-vascular endothelial growth factor (anti-VEGF) agents, as well as pars plana vitrectomy (PPV). However, studies have reported the development of a macular hole (MH) during the course of these treatments [1–7]. Here we report 2 cases of MHs that developed following anti-VEGF therapy and PPV for DME in which the MH showed the formation of a convex surface toward the vitreous cavity, unlike a typical idiopathic MH, and discuss the cause of the MH in each case.

## Case presentations

### Patient 1

**Fig. 1:**
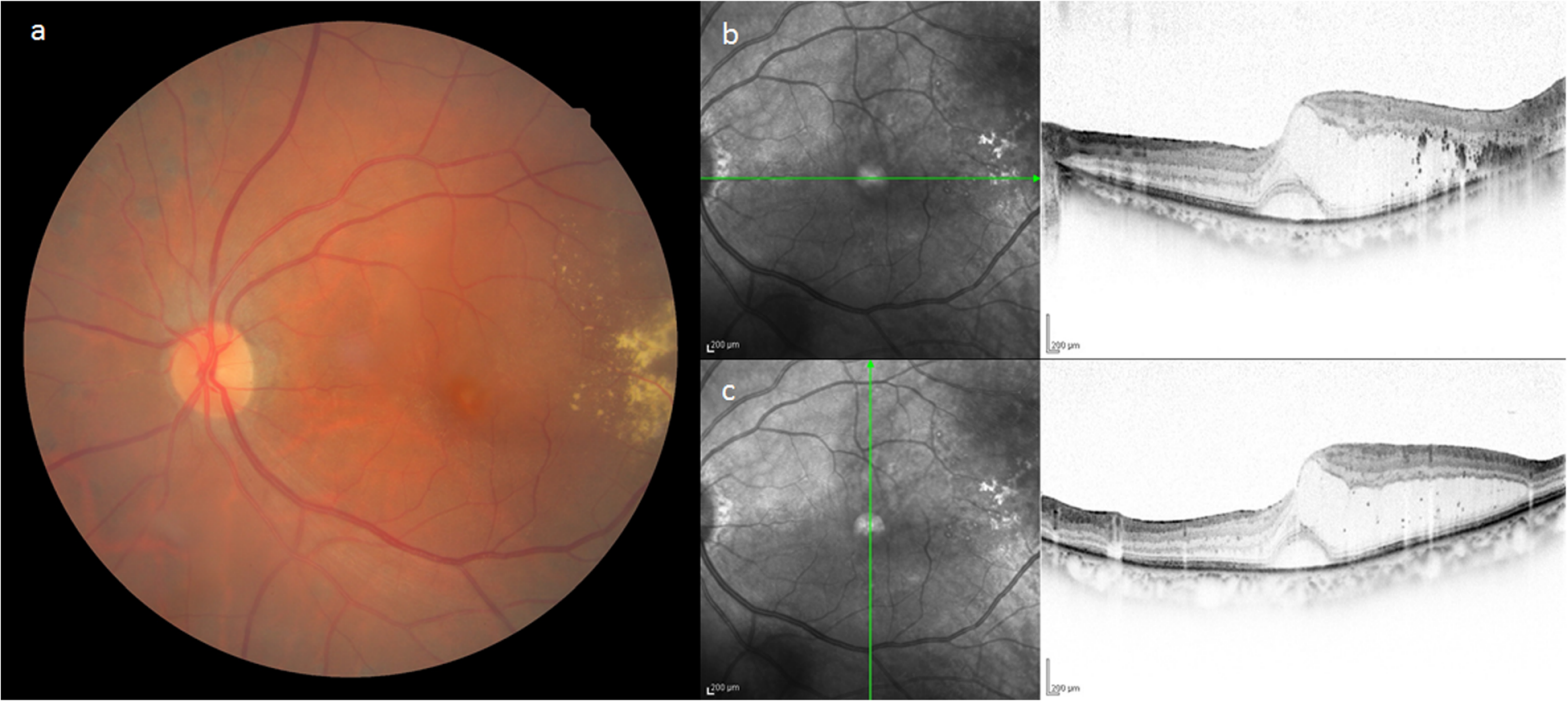
Fundus photograph and optical coherence tomography (OCT) imaging obtained before macular hole (MH) formation in Case 1. The fundus photograph showed hard exudates on the temporal side of the macula, and OCT imaging revealed perifoveal retinoschisis (RS) in addition to diabetic macular edema (DME) and serous retinal detachment (SRD)

Patient 1 was a 73-year-old male who was being followed up after undergoing cataract surgery and panretinal photocoagulation (PRP) for cataract and proliferative diabetic retinopathy (PDR) in his left eye at a nearby hospital. However, in July 2017, the patient was referred to our hospital after being diagnosed with aggravated DME in his left eye. Examination of the patient’s medical history revealed that he had lost vision in his right eye in early childhood, and that his left eye was originally emmetropic. Examination of the patient’s right and left eyes revealed that the corrected visual acuity (VA) was no light perception and 0.6 (log MAR) [non corrigunt (n.c.)], respectively, and that the intraocular pressure (IOP) was 13 mmHg and 14 mmHg, respectively. In the left eye, examination revealed the patient had undergone intraocular lens implantation. The ocular fundus showed hard exudates on the temporal side of the macula, and optical coherence tomography (OCT) examination revealed perifoveal retinoschisis (RS) in addition to DME and serous retinal detachment (SRD) (Fig. 1).

**Fig. 2:**
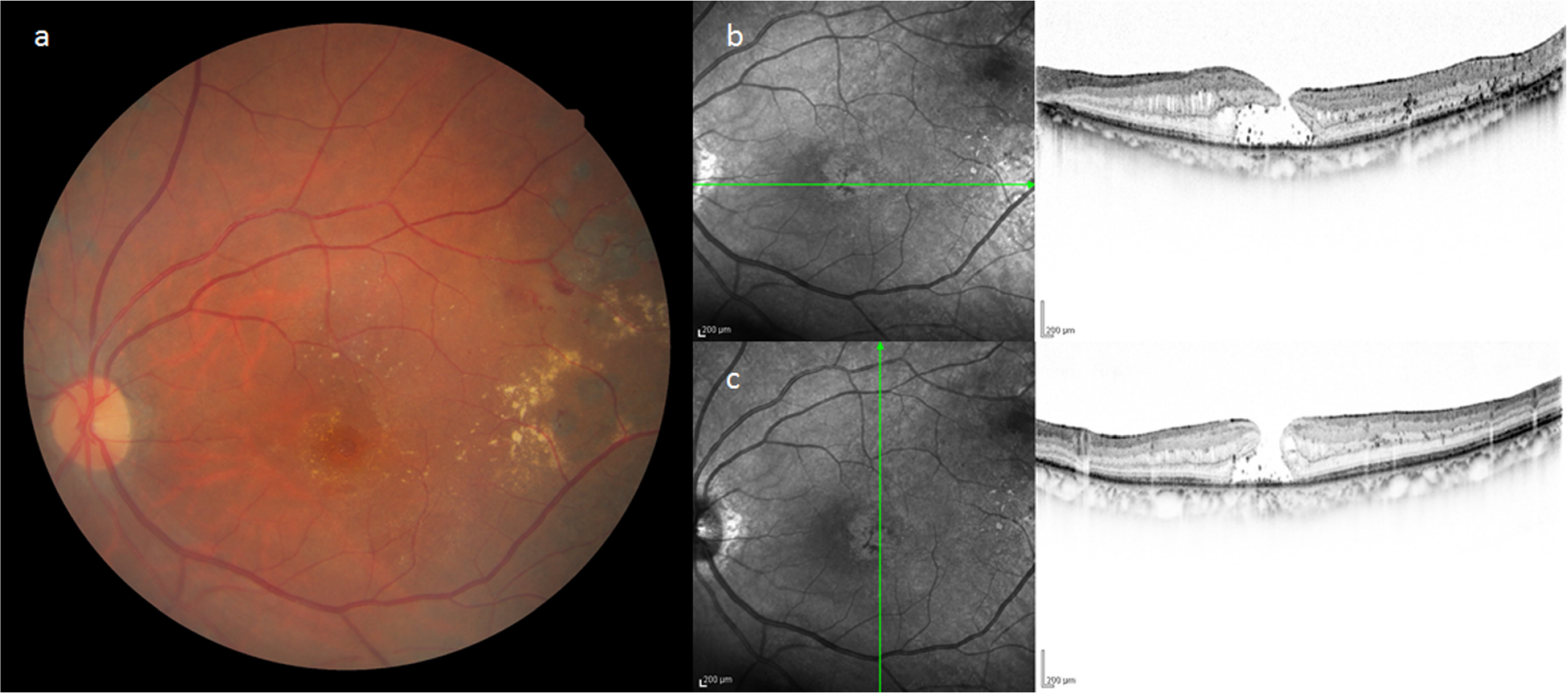
Fundus photograph and OCT imaging obtained at the time of MH formation in Case 1.The hard exudates on the temporal side of the macula gradually increased, resulting in MH in the left eye. Unlike a normal fluid cuff, OCT imaging revealed a convex surface formed at the MH margin toward the vitreous cavity and numerous granular shadows in the fluid cuff

**Fig. 3:**
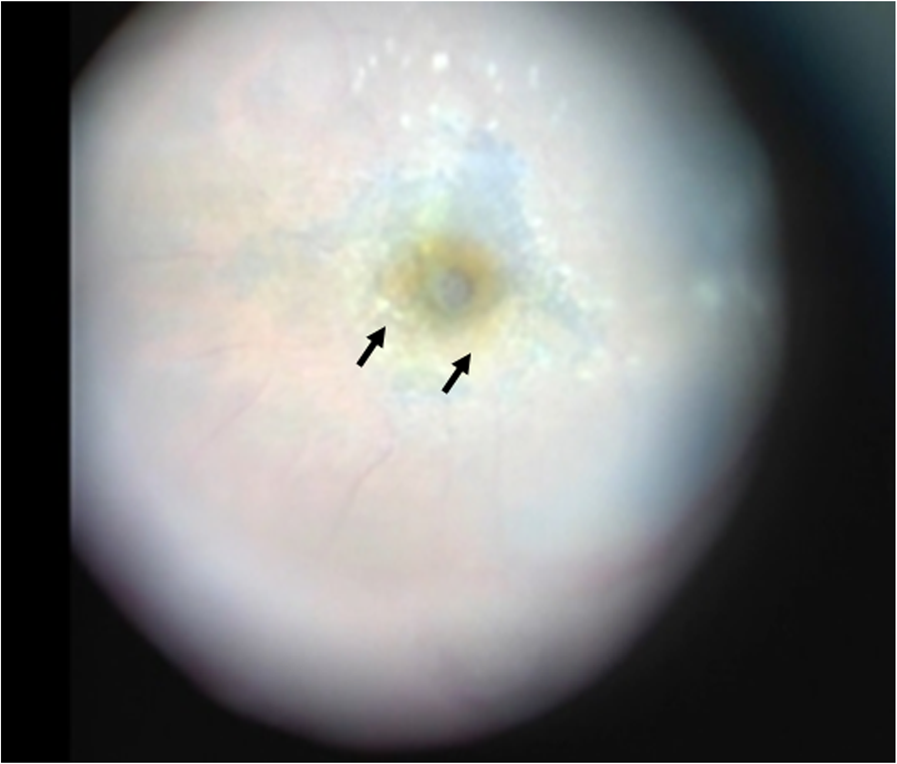
Intraoperative findings in Case 1. The internal limiting membrane was stained with Brilliant Blue G (BBG), however, a thin epiretinal macular membrane not stained with BBG was observed around the MH

**Fig. 4:**
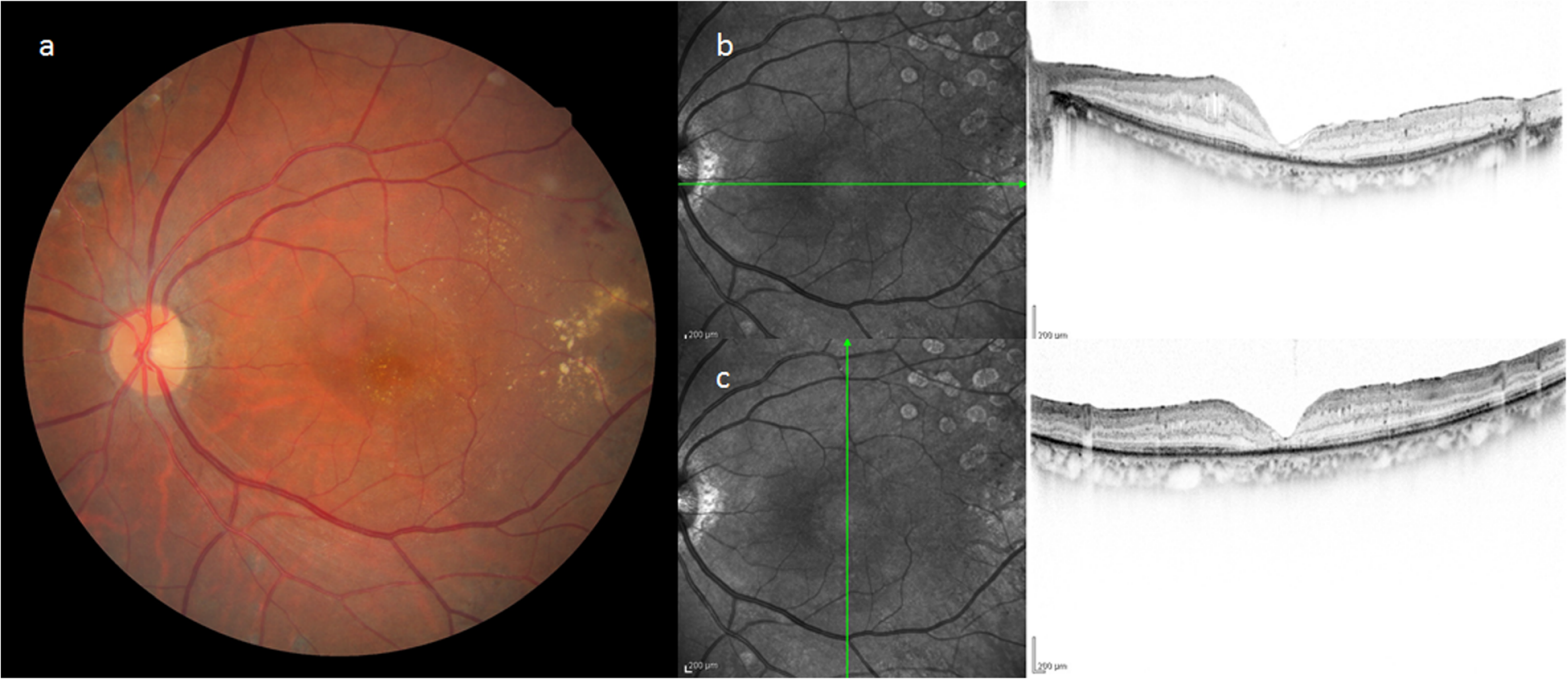
Fundus photograph and OCT imaging obtained after vitreous surgery in Case 1. The MH was closed after surgery, yet the foveal retina became thinner and corrected visual acuity remained at 0.35

For treatment, intravitreal injections of aflibercept was performed four times, yet hard exudates on the temporal side of the macula gradually increased, resulting in the development of an MH (i.e., approximately 300 μm in size) in the left eye in December 2017 (i.e., the same year). Unlike a normal fluid cuff, OCT examination revealed a convex surface was formed at the MH margin toward the vitreous cavity, with numerous granular shadows in the fluid cuff (Fig. 2). The left-eye VA was 0.46 (logMAR). PPV was performed for treatment of the MH. During surgery, the vitreous core was removed, and an artificial posterior vitreous detachment was created from the macular region to the periphery. Subsequently, the internal limiting membrane (ILM) was stained with Brilliant Blue G (BBG), however, a thin epiretinal macular membrane (ERM) not stained with BBG was observed around the MH, which might be secondary effect by anti-VEGF injections (Fig. 3). The ERM was detached together with the ILM, and a concurrent intraocular fluid-air exchange was performed. The patient was then instructed to remain in a prone position directly after surgery. Post surgery, the MH closed, yet the foveal retina became thinner and the corrected VA remained at 0.46 (logMAR) (Fig. 4).

### Patient 2

**Fig. 5:**
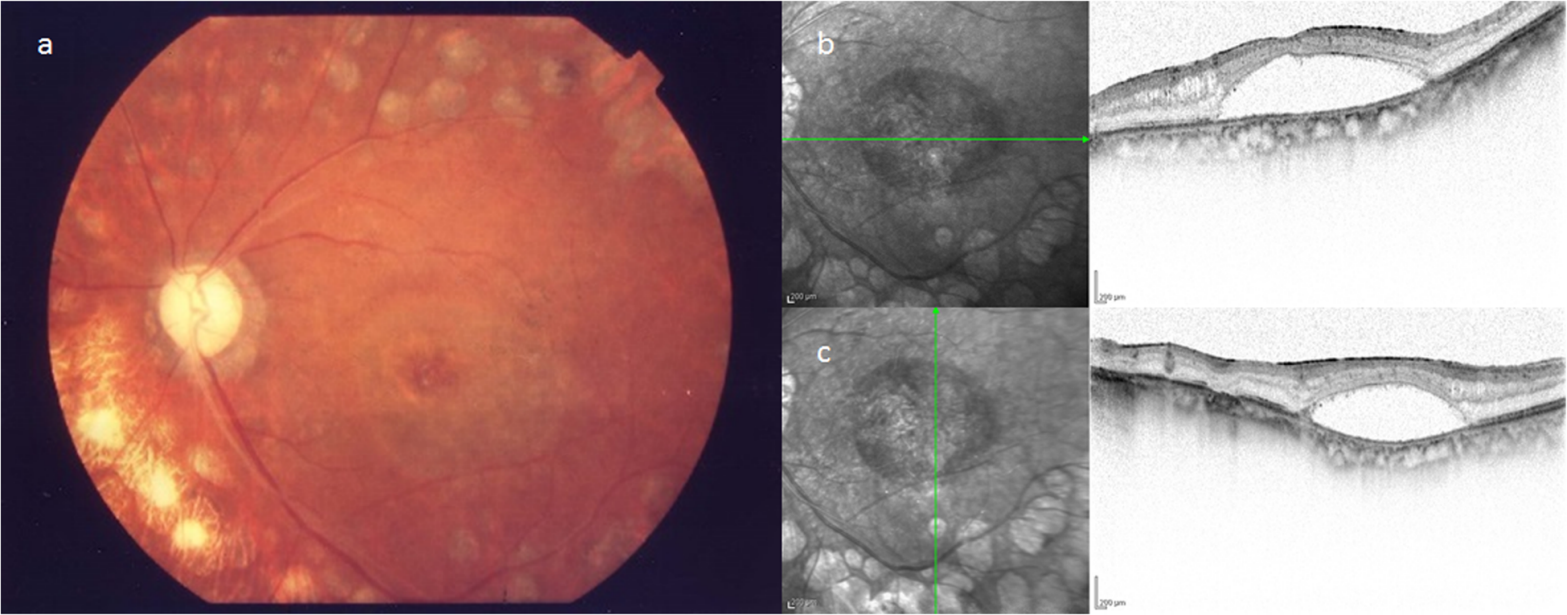
Fundus photograph and OCT imaging obtained before MH formation in Case 2. SRD, RS and ERM (presumably a thickened internal membrane: blue arrows) were partially observed

**Fig. 6:**
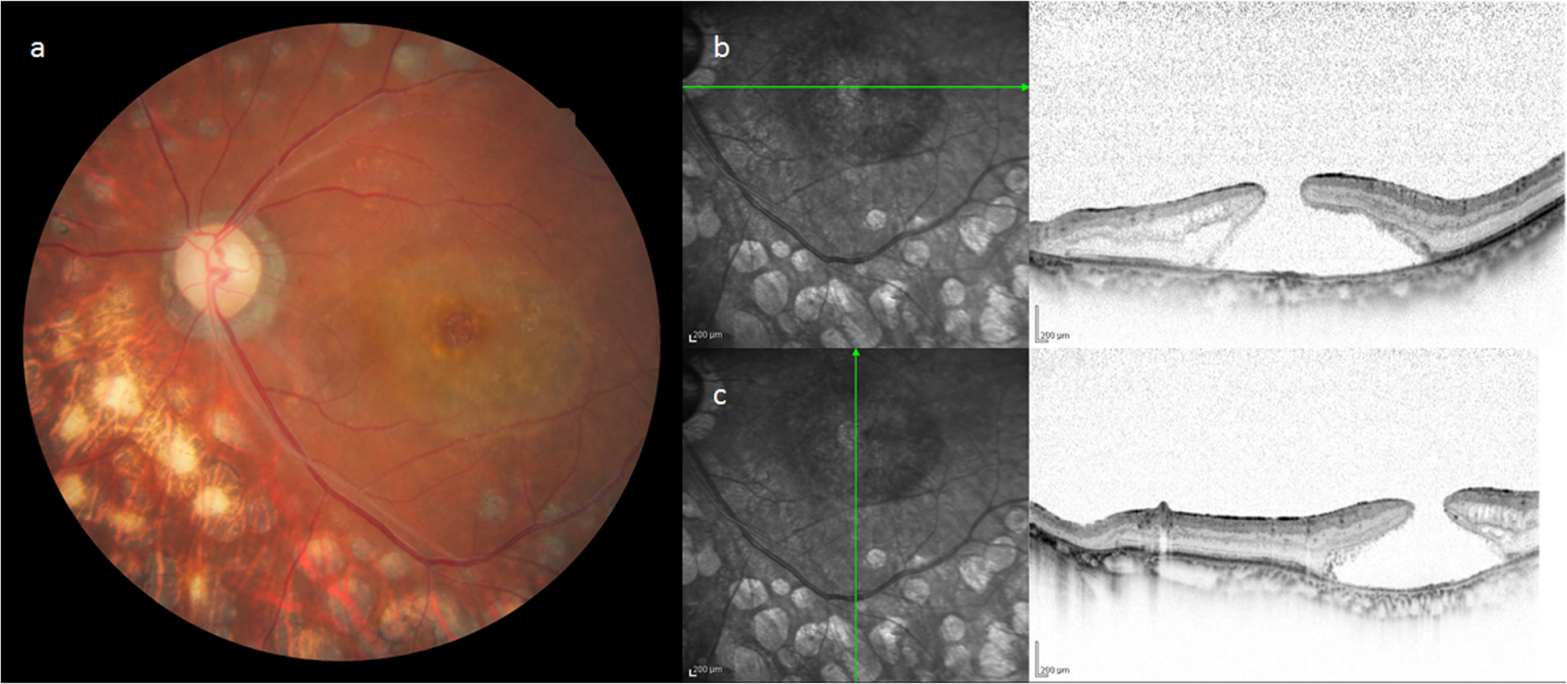
Fundus photograph and OCT imaging obtained at the time of MH formation in Case 2. OCT examination of the left eye revealed residual SRD and ERM (presumably a thickened internal membrane: blue arrows) at the MH margin and the formation of a convex surface toward the vitreous cavity at the retina area of the site, as in Case 1

Patient 2 was a 67-year-old male who was being followed up at a nearby hospital after being diagnosed with PDR and undergoing PRP. In 2006, a vitreous hemorrhage was observed in his left eye, and the patient was subsequently referred to our hospital for surgical treatment. VA at the initial visit was 0.3 (logMAR) (0.16 × S + 3.25 D = C-3.50 × DA 90°) in the right eye and 2.0 (logMAR, n.c.) in the left eye. The IOP was 15 mmHg in both eyes. In January 2007, cataract surgery and vitreous surgery were simultaneously performed in the patient’s left eye. Intraoperative findings showed that the vitreoretinal adhesion was observed entirely in the fundus and artificial posterior vitreous detachment without internal membrane peeling created from the macular region to periphery. Although the visibility of the fundus recovered after surgery, SRD remained, and RS and a thin ERM (presumably a thickened internal membrane: blue aroows in the figure) were partially observed via OCT (Fig. 5). After that, SRD, RS and ERM were persistent, but an MH (i.e., approximately 500 μm in size) developed in the patient’s left eye in June 2012. The corrected VA of the patient’s left eye was 1.6 (logMAR), and OCT examination of that eye revealed residual SRD at the MH margin and the formation of a convex surface toward the vitreous cavity at the retina area of the site, as in Case 1 (Fig. 6). Although vitreous re-surgery was indicated, the patient did not wish to undergo surgery and was thus followed up. Although the MH did not grow during the follow-up course, the degeneration of retinal pigment epithelium progressed and the corrected VA decreased to 2.0 (logMAR) .

## Discussion

Development of an MH during treatment for DME has occasionally been reported. Lecleire-Collet et al. reported a case of concurrent MH and ERM after intravitreal triamcinolone injection for DME; the cause of the MH was the degeneration of Muller cells extended by cystoid macular edema, forming cysts in the retina, yet no retinal vitreous traction was involved [1]. Hasan et al. reported a case of full-thickness MH following anti-VEGF therapy for DME without vitreomacular traction on OCT imaging [2]. However, it should be noted that some studies have reported that vitreous traction may be involved in the cause of MH. Yoon et al. reported a case in which the MH occurred after PPV in combination with peeling of the ILM for DME, thus suggesting the possible involvement of mechanical stress at the time of ILM peeling in the development of MH [3]. Brazitikos et al. reported 8 eyes with an MH following PPV for diabetic retinopathy, 4 of which having concurrent DME. The cause of an MH has been reported to be cystoid changes in the retina due to retinal vitreous traction [4]. Lee et al. reported a case of MH that developed after intravitreal injection of bevacizumab for DME, speculating that traction by the vitreous body incarcerated at the site of insertion of the vitreous injection and change in the properties of the vitreous body by injection are involved as a trigger in the development of the MH [5]. Pessoa et al. performed PPV in 46 eyes of 38 patients with DME with vitreous traction, and reported that an MH developed in 1 eye after surgery [6]. Yamamoto et al. reported that there are two types of MH associated with PDR, i.e., an MH due to traction and an MH due to cystoid macular edema [7]. In Case 1 in this present study, the patient also had an ERM around the MH, thus suggesting the involvement of traction in the tangential direction of the retina in the development of the MH. Taken together, the findings in these reports suggest that it is highly likely that MH secondary to DME develops when retinal vitreous traction is added to the underlying condition, i.e., macular fragility due to long-term edema.

In our two cases, both patients had SRD, RS, and a thin ERM prior to the development of the MH. The primary cause of RS is generally believed to be a fragileity of the layered structure of the retina due to defective Muller cells [8], with the presence of SRD due to DME being considered as its background. It has been reported that diseases such as uveitis, high myopia, and glaucoma can cause RS triggered by SRD [9–11]. In the present two cases, persistent SRD attributable to DME may have caused RS, to which retinal vitreous traction was added, thereby causing the development of the MH. In addition, since the RS caused a fragility of the layered structure of the retina, the retina may have formed a convex surface toward the vitreous cavity, unlike the OCT image seen in typical cases of idiopathic MH. The formation of the convex surface of the retina towards the vitreous body implies that outer layer organization of the retina may have progressed much more than the inner layer organization, thus resulting in a relative decrease in the extensibility of the outer layer, although the details remain unknown.

In Patient 1, the MH was closed with PPV, but the central foveal retinal thickness had markedly decreased and the improvement of VA was poor. This may be due to the fact that the function of the outer layer of the retina, including photoreceptor cells, may have decreased due to the long-term effect of SRD.

It has frequently been reported that PPV is effective for treating MH complicated by DME. Kurihara et al. performed PPV with ILM peeling in 3 patients with MH associated with PDR, and reported that the MH was successfully closed in all patients, thereby leading to resolution of the preoperative DME [12]. On the other hand, some studies have reported that MH closure was possible with pharmacotherapy for DME [13, 14] and that MH closure was spontaneously attained [15]. Thus, conservative treatment may be acceptable as the first modality for MH, especially if the diameter of the MH is small. However, and to the best our knowledge, the atypical MH form observed in our 2 cases is very rare in comparison to the form reported in the previous studies. The persistent DME complicated with SRD, RS, and a thin ERM caused the fragility of the layered structure of the retina, which sometimes might result in the development of the atypical MH formation that was observed in our two cases.

In conclusion, the findings in this study show that an MH secondary to DME has various clinical characteristics that are different from those of a typical idiopathic MH, and that the treatment strategy should be determined after fully understanding the pathological condition.

## Data Availability

The datasets during the current study are available from the corresponding author on reasonable request.

## Abbreviations

MH: Macular hole
DME: Diabetic macular edema
PPV: Pars plana vitrectomy
RS: Retinoschisis
SRD: Serous retinal detachment
IOP: Intraocular pressure
BBG: Brilliant Blue G
